# Differential effects of sound interventions tuned to 432 Hz or 443 Hz on cardiovascular parameters in cancer patients: a randomized cross-over trial

**DOI:** 10.1186/s12906-025-04758-5

**Published:** 2025-01-22

**Authors:** Anna Hohneck, Ánxelo Maia Rodríguez, Simone Weingärtner, Kirsten Merx, Felicitas Sarodnick, Fritjof von Gagern, Athanasios Mavratzas, Iris Burkholder, Gerhard Schumacher, Wolf-Karsten Hofmann, Ralf-Dieter Hofheinz

**Affiliations:** 1 Department of Cardiology, Angiology, Haemostaseology and Medical Intensive Care, University Medical Centre Mannheim, Medical Faculty Mannheim, Heidelberg University, Mannheim, Germany; 2 European Center for AngioScience (ECAS) and German Center for Cardiovascular Research (DZHK) Partner Site Heidelberg/Mannheim, Mannheim, Germany; 3 Department of Hematology and Oncology, University Medical Centre Mannheim, Heidelberg University, Mannheim, Germany; 4 Mannheim Cancer Center, Mannheim, Germany; 5 Musikalische Akademie Mannheim e.V., Mannheim, Germany; 6 Department of Obstetrics and Gynecology, Medical Faculty Mannheim, University Medical Centre Mannheim, Heidelberg University, Mannheim, Germany; 7 Department of Nursing and Health, University of Applied Sciences of the Saarland, Saarbruecken, Germany; 8 inmediQ GmbH, Butzbach, Germany

**Keywords:** Sound intervention, Tuning, Cardiovascular parameters, Concert pitch

## Abstract

**Background:**

This study investigated whether a sound intervention tuned to 432 Hz (Hz) yields differential effects on cardiovascular parameters and psychological outcomes compared to 443 Hz, which is the concert pitch in German professional orchestras.

**Methods:**

Using a randomized cross-over design, patients with cancer were recruited to receive both a 15-minute sound intervention with a body monochord tuned to 432–443 Hz. Before (*pre*) and after (*post*) intervention, cardiovascular parameters were measured using the VascAssist2.0. In addition, visual analogue scales (VAS) for emotional well-being, anxiety, stress, pain and sadness were also assessed *pre* and *post* intervention.

**Results:**

43 patients (8 male, 35 female) with a median age of 61 years (range 35–86) were included. Both interventions led to a significant reduction in heart rate with a more pronounced effect for 432 Hz (median reduction − 3 bpm (432 Hz) vs. median reduction − 1 bpm (443 Hz), *p* = 0.04). While heart rate variability was increased exclusively by 432 Hz (median increase + 3 ms, *p* = 0.01), both vascular resistance (median reduction − 5%, *p* = 0.008) and stiffness (median reduction %, *p* = 0.04) were significantly reduced by 432 Hz, which was not observed at 443 Hz. Nevertheless, these effects were not significantly different compared to 443 Hz. On the other hand, 432 Hz led to a reduced pulse wave velocity (median reduction − 0.5 m/s, *p* < 0.001), which was also significantly different compared to 443 Hz (*p* < 0.001). Improvement in VAS was observed for both groups, with significant increases in emotional well-being and reduction in fatigue, anxiety and stress for both intervention timepoints, although the majority showed no increased VAS scores even before the intervention (median values 0 for anxiety and stress).

**Conclusion:**

Sound interventions tuned to 432–443 Hz exert both positive effects in cancer patients. While psychological outcomes are improved by both interventions, 432 Hz leads to a more pronounced but not significantly different effect to 443 Hz on objective cardiovascular parameters, which reflect deeper relaxation.

**Supplementary Information:**

The online version contains supplementary material available at 10.1186/s12906-025-04758-5.

## Introduction

The concept of using 432 Hz as a concert pitch for musical instruments has garnered significant attention due to its purported positive effects on both physical and mental well-being [1]. In contrast to the standard concert pitch of 440 Hz, which was established at the 1939 International Conference in London, the 432 Hz tuning is advocated by some as a more natural and harmonious alternative [2]. However, the theoretical basis for the alleged natural physiological effects of 432 Hz, such as a certain vibration ratio that is in harmony with the natural vibration of the human body, is based on historical and philosophical interpretations and not on empirical data. For instance, the scientist and founder of acoustics, Joseph Sauveur, and other advocates suggested a musical basis where the note C1 (“middle C”) is at 256 Hz, corresponding to the concert pitch of A1 = 432 Hz [3]. This theory is based on the principle that doubling the frequency leads to an octave increase in tone. Today, otologists and neurologists still use tuning forks that are tuned to c = 128 Hz or c^1^ = 256 Hz [4]. Nevertheless, relaxing effects can be attributed to 432 Hz music, which is why it is often incorporated into music therapy or music medicine practice [5, 6]. Music therapy and music medicine are two distinct approaches that utilize music in therapeutic settings, while the focus of music therapy lies on the therapeutic relationship and is carried out by a certified music therapist, and music medicine refers to the use of pre-recorded music as a complementary treatment administered by healthcare professionals [7]. As the transitions are fluid, it is not always possible to make an exact distinction between music therapy and music medicine, especially between receptive music therapy and music medicine [8]. Recent studies have investigated the physiological and psychological effects of music tuned to 432 Hz compared to the standard 440 Hz. A cross-over pilot study conducted in 2019 found that listening to music at 432 Hz resulted in a marked decrease in heart rate and slight decreases in blood pressure and respiratory rate, compared to 440 Hz tuning [5]. Additionally, participants reported higher levels of focus and satisfaction after listening to the 432 Hz music. Another study examined the effects of 432 Hz music on the sleep quality of patients with spinal cord injuries. The results showed a significant improvement in sleep quality when patients listened to their favorite music tuned to 432 Hz, as opposed to 440 Hz [9]. Regarding emotional mood, 432 Hz music led to significant improvement, compared to 440 Hz, particularly in male participants, while heart rate was not affected [10]. While these studies suggest potential benefits of 432 Hz tuning, it is crucial to acknowledge the limitations and gaps in the current evidence base. There is a need for more extensive, randomized controlled trials to fully validate the therapeutic effects of 432 Hz tuning. The present study sought to assess the differential effects of a receptive music therapy with sound interventions with a body monochord tuned to 432–443 Hz on cardiovascular parameters and psychological outcomes in cancer patients.

## Methods

### Study design and setting

This randomized cross-over study was performed at the University Medical Centre Mannheim, University of Heidelberg, Germany. It was designed as a head-to-head comparison of a sound intervention tuned to 432–443 Hz. Recruitment started in June 2023 and ended in October 2023. Patients were not followed up.

The study was conducted according to the principles of the declaration of Helsinki and was approved by the local ethical committee, Medical Ethics Commission II, Faculty of Medicine Mannheim, University of Heidelberg, Germany (2019–763 N). Data protection was in accordance with the EU Data Protection Directive.

## Materials and methods

### Study population

Forty-five individuals > 18 years were included and gave written informed consent. Exclusion criteria comprised serious hearing impairment, acute coronary syndrome or stroke within four weeks before study participation, planned bypass surgery, dependence on pacemaker, cardiac arrhythmia (e.g., atrial fibrillation, sick-sinus-syndrome, sinoatrial/ atrioventricular block), uncontrolled hypertension or severely low blood pressure (< 90/50 mmHg) and breast cancer with bilateral axillary dissection.

### Baseline characteristics

Baseline demographic characteristics (age, gender, weight, height, etc.) were collected from all study participants, as well as information on existing medication and physical activity. In addition, participants were asked if they were married and had children. Preferred music genre (multiple answers possible) was assessed and it was questioned whether there is a hearing loss.

In addition, the Spielberger State-Trait Anxiety Inventory (STAI) [11], which is a psychometric test consisting of 40 self-report items on a four-point Likert scale, and the European Organization for Research and Treatment of Cancer (EORTC) Quality of Life Questionnaire (QLQ-C30) [12] were assessed at baseline. The STAI measures two types of anxiety – state anxiety and trait anxiety. Higher scores are positively correlated with higher levels of anxiety. The QLQ-C30 is composed of 30 questions, comprising 15 subscales on three domains (global health status, functional scales, and symptom scales), with a score ranging from 0 to 100 points for all subscales. A higher score in global health status or functional scales represents a higher quality of life or better level of functioning. However, in the symptom subscales, a higher score represents a higher level of symptoms or problems.

### Sound intervention

All patients received both a 15-minute sound intervention with a body monochord tuned to a concert pitch of 432–443 Hz as part of a cross-over design. Interventions were performed in lying position after a 10-minute resting period. In order to rule out a systematic bias, the sequence of intervention was randomized. The two interventions took place within four weeks with at least a one-week blanking period between the two interventions. The sound intervention was performed with the body monochord called “Heaven and Earth” (http://klangkoerper.de/himmel-und-erde.html) [13]. This instrument consists of a semi-open resonance body with 29 strings (24 of those in C1sharp, 2 in C2sharp, 2 C3sharp, and 1 in G3sharp) and is an established method in everyday clinical practice for music therapy [14, 15]. The monochord was placed on the chest during the intervention to ensure optimal transmission of the vibrations. The instrument was played for 15 min by one of the investigators (AMR) in all patients.

### Cardiovascular parameters

Cardiovascular parameters were assessed non-invasively using the VascAssist2.0 device (inmediQ GmbH, Butzbach, Germany) after a 10-minute resting phase before (*pre*) and after (*post*) the intervention and evaluated both for the total population and separately according to the intervention type (432 Hz vs. 443 Hz). A detailed measurement protocol can be found in the appendix.

The VascAssist2.0 is an oscillometric device that evaluates vascular properties non-invasively using model-based pulse wave analysis. In addition to vital parameters (heart rate, brachial blood pressure), pulse wave velocity (PWV), as a direct measure of arterial stiffness, and vascular indices (resistance, stiffness), can be determined. Vascular age as a combined variable can be determined from these vascular indices and the chronological age [16]. Beyond that, augmentation index (Aix; also a marker for arterial stiffness and determinant of central hemodynamics) and left ventricular ejection time (LVET, indicator of systolic length) are also provided. Moreover, the root mean square of successive differences between normal heartbeats (RMSSD) as heart rate variability (HRV) metric and stress indicator is captured [17].

### Psychological outcomes and personal assessment

Visual analogue scales (VAS) for emotional well-being, anxiety, stress, pain and sadness were assessed before (*pre*) and after (*post*) the intervention. The VAS indicates a continuum ranging from 0 to 100, where 0 represents the minimal and 100 the maximal extent. Moreover, after completing the two interventions, the study participants were asked whether they had noticed a difference between the sessions and which session they preferred.

### Statistical analysis

Quantitative characteristics were evaluated using statistical parameters (N, arithmetic mean, standard deviation, median, minimum and maximum) and qualitative characteristics were evaluated in the form of frequency tables.

Cardiovascular parameters were measured before and after sound intervention (432–443 Hz) and evaluated descriptively both for the total population and per measurement time. In addition, the differences to the baseline value were calculated and also analyzed descriptively, using the Wilcoxon signed-rank test. The results were visualized with the help of grouped boxplots per parameter and measurement time and waterfall plots to display intraindividual differences before and after the intervention.

Psychological outcomes were evaluated in the same way as cardiovascular parameters. In addition, scatter plots of the measurements before and after the intervention were created grouped according to the type of intervention.

All statistical analyses were performed with SAS 9.4. (SAS Institute Inc., North Carolina). Statistical significance was assumed at *p* < 0.05. The comparison of the two interventions is of exploratory nature.

## Results

A total of 45 patients were included in the study, whereby data from 43 patients were included in the final analysis (as two patients did not attend the second visit).

### Baseline characteristics

Baseline characteristics are shown in Table 1.

**Table 1:** Baseline characteristics

	All patients (*N* = 43)	432–443 Hz (*n* = 22)	443 Hz–432 Hz (*n* = 21)
Sex, female (%)	35 (81)	16 (73)	19 (91)
Age, years	61 (35–86)	59 (35–86)	62 (37–80)
BMI (kg/m^2^)	24 (17–38)	24 (17–37)	25 (19–38)
Married, yes (%)	23 (54)	12 (55)	11 (52)
Children, yes (%)	35 (81)	17 (77)	18 (86)
Regular physical activity (%)	16 (37)	9 (41)	7 (33)
Hearing loss (%)	0 (0)	-	-
Preferred music genre (%) (multiple responses possible)			
Pop	22 (51)	12 (55)	10 (48)
Rock	19 (44)	8 (36)	11 (52)
Jazz	14 (33)	4 (18)	10 (48)
Classical music	22 (51)	10 (46)	12 (57)
Hits	11 (26)	7 (32)	4 (19)
Hip Hop	2 (4.7)	1 (4.5)	1 (4.8)
Others	13 (20)	8 (36)	5 (24)
**QLQ-C30**, median (range)			
*Global health status*	67 (0–100)	67 (0–100)	67 (25–88)
*Functional scales*			
physical	73 (33–100)	73 (33–100)	73 (53–100)
role	67 (0–100)	67 (0–100)	67 (0–100)
cognitive	67 (17–100)	67 (17–100)	67 (33–100)
emotional	58 (17–100)	58 (17–100)	67 (17–100)
social	67 (0–100)	67 (0–100)	67 (0–100)
*Symptom scales*			
fatigue	44 (0–100)	44 (0–100)	33 (11–89)
pain	33 (0–83)	33 (0–83)	33 (0–83)
nausea/vomiting	0 (0–100)	0 (0–50)	0 (0–100)
Dyspnoea	33 (0–100)	33 (0–100)	33 (0–100)
Insomnia	50 (0–100)	67 (0–100)	33 (0–100)
Appetite loss	0 (0–100)	0 (0–67)	0 (0–100)
Constipation	0 (0–100)	0 (0–100)	0 (0–100)
Diarrhoea	0 (0–100)	0 (0–100)	0 (0–100)
Financial difficulties	0 (0–100)	0 (0–100)	0 (0–100)
**STAI**, median (range)	44 (27–60)	48 (28–55)	43 (27–60)

Data are presented as median (range) or numbers (frequencies)

BMI, body mass index; QLQ-C30, European Organisation for Research and Treatment of Cancer (EORTC) Quality of Life Questionnaire (QLQ) composed of 30 questions; STAI, state trait anxiety inventory

A total of 8 patients were male (19%) and 35 patients (81%) were female. The median age was 61 years (range, 35–86 years). 23 patients (54%) were married and 35 patients (81%) reported having children. Regular physical activity was practiced by 16 study participants (37%). Data on cancer type, stages and treatment can be obtained from Supplemental Table 1 (ST 1).

Most common preferred music genres were pop (22 pts, 51%) and classical music (22 pts, 51%), followed by rock (19 pts, 44%) and jazz (14 pts, 33%).

Median values for global health status, assessed using the QLQ-C30, were 66.7 points (0–100), with median values of 58 to 73 observed for the functional scales and symptom scales ranging from a median of 0 to 50. Median STAI scores, to reflect anxiety levels, were 44 (27–60), and thus slightly above the average for the German population [18].

### Cardiovascular parameters

Cardiovascular parameters were assessed using the VascAssist before (pre) and after (post) the sound intervention (Table 2).

**Table 2:** Cardiovascular parameters

	432 Hz (*n* = 43)	443 Hz (*n* = 43)	*p*-value (Wilcoxon signed-rank test)
**Heart rate (bpm)**			
pre	71 (51–94)	69 (47–109)	0.59
post	67 (50–90)	68 (51–100)	0.32
Difference post - pre	-3 (-15–4)	-1 (-12–9)	0.04
p-value	< 0.001	0.01	
**Brachial systolic BP (mmHg)**			
pre	127 (103–166)	125 (97–162)	0.98
post	120 (92–158)	123 (95–158)	0.46
Difference post - pre	-4 (-25–10)	-3 (-23–5)	0.48
p-value	< 0.001	< 0.001	
**Brachial diastolic BP (mmHg)**			
pre	71 (52–92)	70 (48–91)	0.69
post	66 (49–89)	68 (44–91)	0.12
Difference post - pre	-3 (-23–9)	0 (-15–25)	0.08
p-value	0.001	0.37	
**RMSSD (ms)**			
pre	21 (7–192)	24 (6–175)	0.52
post	27 (9–163)	25 (6–173)	0.21
Difference post - pre	3 (-29–42)	1 (-48–31)	0.17
p-value	0.01	0.40	
**Resistance (%)**			
pre	63 (15–114)	56 (14–124)	0.96
post	54 (15–120)	55 (21–124)	0.28
Difference post - pre	-5 (-38–18)	-3 (-21–37)	0.18
p-value	0.008	0.29	
**Stiffness (%)**			
pre	60 (25–101)	58 (21–95)	0.52
post	49 (15–91)	59 (15–95)	0.04
Difference post - pre	-5 (-28–23)	-2 (-29–24)	0.49
p-value	0.04	0.21	
**Aortic systolic BP (mmHg)**			
pre	106 (87–153)	109 (83–155)	0.86
post	105 (81–149)	110 (80–149)	0.55
Difference post - pre	-4 (-22–6)	-3 (-25–7)	0.65
p-value	< 0.001	0.002	
**Aortic diastolic BP (mmHg)**			
pre	72 (54–94)	72 (50–93)	0.91
post	66 (52–91)	70 (46–92)	0.09
Difference post - pre	-3 (-22–9)	-1 (-14–12)	0.07
p-value	< 0.001	0.10	
**Aix@75 (%)**			
pre	16 (-10–84)	16 (-16–92)	0.52
post	15 (-8–92)	16 (-14–43)	0.92
Difference post - pre	-0.8 (-20–9)	0.4 (-10–6)	0.64
p-value	0.68	0.85	
**Aortic PWV (m/s)**			
pre	9 (6–15)	8 (4–18)	0.003
post	8 (6–16)	8 (4–19)	0.29
Difference post - pre	-0.5 (-5–7)	0 (-15–11)	< 0.001
p-value	< 0.001	0.70	
**LVET (ms)**			
pre	270 (207–335)	267 (199–344)	0.99
post	272 (227–350)	271 (193–322)	0.49
Difference post - pre	6 (-32–62)	4 (-30–62)	0.19
p-value	< 0.001	0.06	
**Vascular age**			
pre	61 (28–89)	60 (33–94)	0.77
post	57 (25–92)	56 (31–95)	0.25
Difference post - pre	-3 (-16–12)	-1 (-8–17)	0.11
p-value	0.002	0.20	

Data are presented as median (range)

Aix@75, augmentation index standardized to a heart rate of 75 bpm (beats per minute); BP, blood pressure; LVET, left ventricular ejection time; PWV, pulse wave velocity; RMSSD, root mean square of successive difference

Note: comparisons between groups and measurement time point are performed using the Wilcoxon signed rank sum test

#### Vitals

**Fig. 1:**
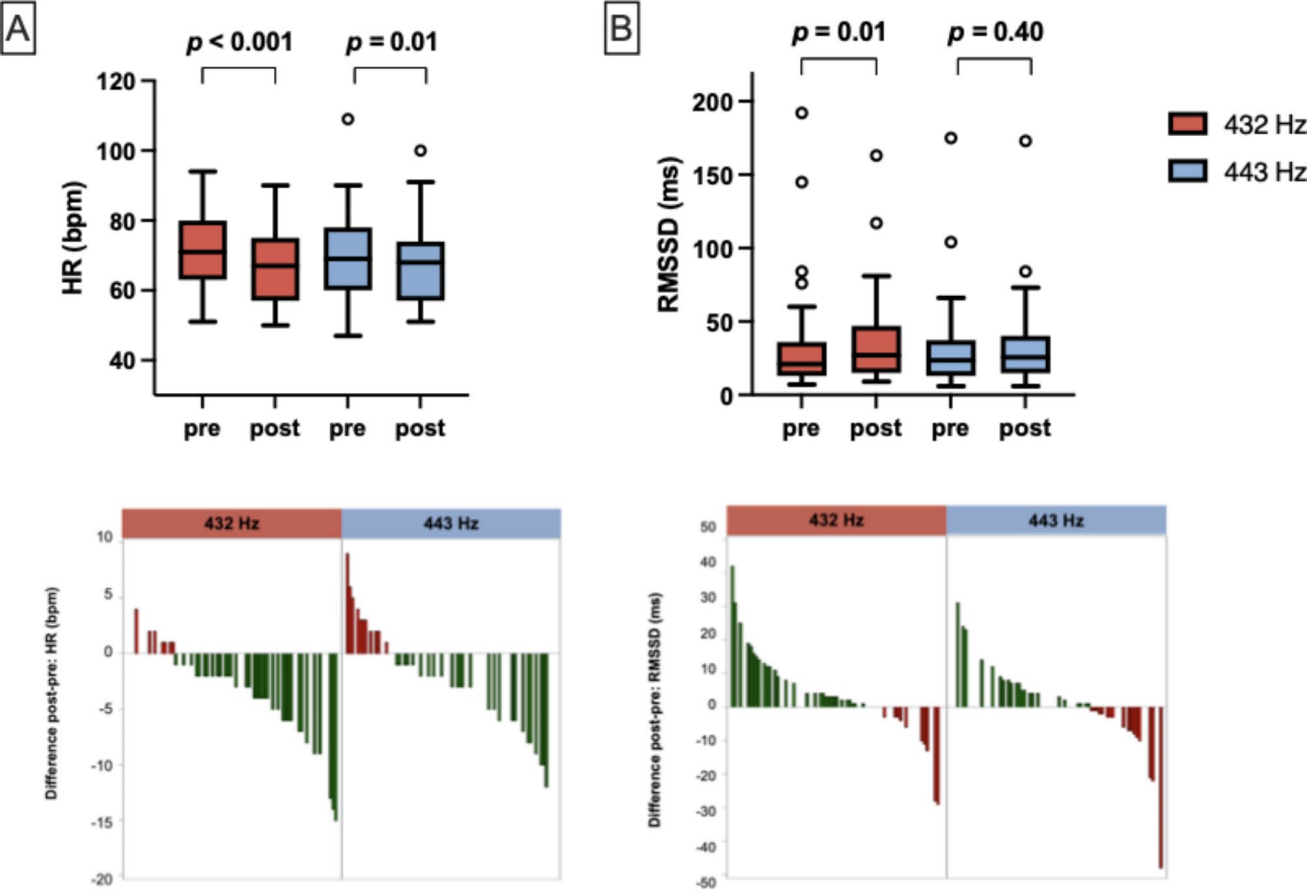
Vitals. Grouped boxplots for both 432 Hz (red) and 443 Hz (blue) intervention and waterfall plots displaying intraindividual difference before (pre) and after (post) the intervention for (**a**) HR (heart rate) and (**b**) RMSSD (root mean square of successive differences) as HRV (heart rate variability) measure. While both interventions led to a HR reduction (with a more pronounced effect for 432 Hz), HRV was increased exclusively by 432 Hz

Both interventions led to a significant reduction in heart rate (432 Hz *p* < 0.001; 443 Hz *p* = 0.01), with a more pronounced effect for 432 Hz (median reduction − 3 bpm (432 Hz) vs. median reduction − 1 bpm (443 Hz), *p* = 0.04) (Fig. 1a). Systolic blood pressure was also reduced by both interventions (*p* < 0.001), while diastolic blood pressure did not change. HRV, on the other hand, measured as RMSSD (ms) was increased exclusively by 432 Hz (median increase + 3 ms, *p* = 0.01; 432 Hz vs. 443: *p* = 0.17) (Fig. 1b).

#### Vascular indices

**Fig. 2:**
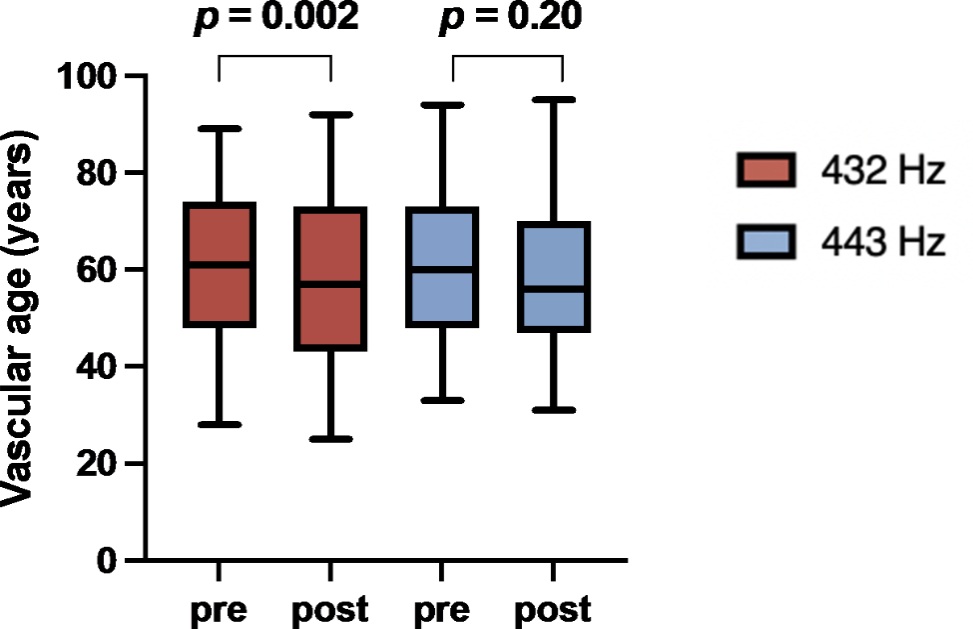
Vascular age. Grouped boxplots for both 432 Hz (red) and 443 Hz (blue) intervention before (pre) and after (post) the intervention. While 432 Hz significantly reduced vascular age, there was no change for 443 Hz

The 432 Hz intervention exerted effects on vascular indices, which were not observed at 443 Hz. Both resistance (median reduction − 5%, *p* = 0.008; 432 Hz vs. 443 Hz: *p* = 0.18) and stiffness (median reduction %, *p* = 0.04; 432 Hz vs. 443 Hz: *p* = 0.49) were significantly reduced by 432 Hz, with a median decrease of 3 years in vascular age (pre median 61 years (28–89) vs. post median 57 years (25–92), *p* = 0.002; 432 Hz vs. 443 Hz: *p* = 0.11) (Fig. 2).

#### Central hemodynamics

Effects on aortic blood pressure after the interventions were also detected. While both interventions led to a reduction in systolic aortic blood pressure (432 Hz median reduction − 4 mmHg *p* < 0.001; 443 Hz median reduction − 3 mmHg, *p* = 0.002; 432 Hz vs. 443 Hz: *p* = 0.65), changes in diastolic aortic blood pressure were only observed for 432 Hz (median reduction − 3 mmHg, *p* < 0.001), with a trend towards a statistical difference compared to 443 Hz (*p* = 0.07). Aix remained unaffected. PWV was significantly reduced by 432 Hz (median reduction − 0.5 m/s, *p* < 0.001), which was also significantly different compared to 443 Hz (*p* < 0.001). However, it should be noted that the PWV in the 432 Hz group was significantly higher at baseline than in the 443 Hz group (PWV (m/s) 432 Hz median 9 (6–15) vs. 443 Hz median 8 (4–18), *p* = 0.003). LVET, as indicator of systolic length, was extended by both interventions (432 Hz median increase + 6 ms, *p* < 0.001, 443 Hz median increase + 4 ms, *p* = 0.06), but failed to reach statistical significance for 443 Hz.

### Psychological outcomes

An improvement in psychological outcomes was observed in almost all study participants for both sound interventions. This was particularly evident in an increase in emotional well-being (*p* < 0.001 for both interventions). In addition, both interventions led to a reduction in fatigue, with a more pronounced effect for 432 Hz (432 Hz *p* = 0.005; 443 Hz *p* = 0.01). Furthermore, an improvement in anxiety and stress was also achieved in a large number of study participants (VAS anxiety 432 Hz *p* = 0.008, 443 Hz *p* < 0.001; VAS stress *p* < 0.001 for both interventions), although the majority showed no increased VAS scores even before the intervention (median values 0 for anxiety and stress). No statistical difference was found between the effects of the two groups (Table 3).

**Table 3:** Psychological outcomes

	432 Hz (*n* = 43)	443 Hz (*n* = 43)	*p*-value (Wilcoxon signed-rank test)
**VAS Emotional well-being**			
pre	69 (20–100)	71 (26–100)	0.83
post	78 (35–100)	75 (15–100)	0.64
Difference post - pre	6 (0–50)	3 (-37–31)	0.55
p-value	< 0.001	< 0.001	
**VAS Anxiety**			
pre	0 (0–76)	0 (0–75)	0.22
post	0 (0–6)	0 (0–60)	0.006
Difference post - pre	0 (-76–0)	0 (-75–0)	0.64
p-value	0.008	< 0.001	
**VAS Stress**			
pre	0 (0–79)	0 (0–85)	0.66
post	0 (0–77)	0 (0–100)	0.99
Difference post - pre	0 (-47–0)	0 (-61–15)	0.42
p-value	< 0.001	< 0.001	
**VAS Fatigue**			
pre	13 (0–100)	28 (0–100)	0.16
post	0 (0–98)	2 (0–73)	0.07
Difference post - pre	0 (-84–18)	0 (-92–45)	0.97
p-value	0.005	0.01	

Data are presented as median (range)

STAI: state trait anxiety inventory; VAS: visual analogue scale

Note: the two groups are compared using the Mann-Whitney-U test while the change between the measurement time points is compared using the Wilcoxon signed rank sum test. Visual analogue scales ranged from 0 to 100

### Personal assessment

Twenty-eight patients (65%) reported having noticed a difference between the two interventions. 24 patients (56%) preferred the 432 Hz sound intervention while six patients (14%) appreciated the 443 Hz sound intervention. Almost a third of patients (13 patients, 30%) stated that they liked both interventions equally.

## Discussion

There are only a few studies to date that investigate the effect of 432 Hz music, four of which compare 432 Hz with the international standard pitch of 440 Hz. The present study compared 432 Hz with 443 Hz, which is the concert pitch in German professional orchestras. We chose 443 Hz to maximize the pitch difference between the two frequencies. This pitch difference, which would be roughly equivalent to a “quarter tone”, is not noticeable to the average listener. Individuals with absolute perception (“absolute pitch” or so-called absolute listeners) may experience difficulties with 432 Hz music, as they cannot reliably assign the tones they hear to a pitch. Absolute pitch is the ability to perceive pitch class and to mentally categorize sounds according to this perceived pitch class [19]. It could therefore be that 432 Hz music has less of an effect on absolute listeners. The incidence of absolute pitch is given as 1:10.000, whereby professional musicians are significantly more likely to be absolute listeners [20]. This supports the hypothesis that absolute pitch is not an “innate gift”, but a memory performance that can be achieved through regular training [21]. It might therefore be interesting to investigate whether sound interventions in general and 432 Hz in particular have the same beneficial effects in professional musicians. A study from 2022 revealed that music tuned to 432 Hz resulted in higher perceived arousal, assessed by personal rating [22]. In this context, it would have been interesting to also explore objective measures. One advantage of our study is that both personal ratings (VAS) and objective measures (cardiovascular parameters) were assessed. While both interventions led to an improvement in psychological outcomes (with no relevant difference), beneficial effects on cardiovascular parameters were observed, which were more pronounced at 432 Hz than at 443 Hz. For example, a greater reduction in heart rate and blood pressure was observed at 432 Hz, which is in line with data from Calamassi et al. [23, 24]. In addition, heart rate variability, which has prognostic implications in patients with cancer, was increased exclusively by 432 Hz [25, 26]. The best-established parameter for vascular stiffness is pulse wave velocity (PWV). PWV indicates the propagation velocity at which the blood pressure pulse propagates through the circulatory system [27]. PWV is an important diagnostic marker for the assessment of vascular stiffness and is used as a predictor for cardiovascular events [28]. The vascular resistance and the arterial wall properties largely determine the speed of the PWV. Both vascular resistance and stiffness were significantly reduced by 432 Hz, which was also reflected in a decrease in PWV. This is congruent with results from earlier studies by our group [29, 30] and further studies that investigated the effects of music on arterial stiffness [31, 32]. However, it should be noted that the PWV in the 432 Hz group was significantly higher at baseline than in the 443 Hz group.

At first glance, one might speculate that patients allocated to the 432 Hz group tended to be sicker and therefore concluded that they benefited more from the sound intervention. However, the choice of the cross-over design argues against this potential bias. In addition, a decrease in vascular age as a combined variable could also only be observed by 432 Hz. Vascular age is often used in cardiology to communicate cardiovascular risk because of its emotional character [33]. On the other hand, it breaks down the results of the present study to a single number: a 432 Hz intervention reduced vascular age by a median of 3 years! Furthermore, effects on central hemodynamics could be demonstrated. While 432 Hz reduced both systolic and diastolic aortic blood pressure, for 443 Hz only a reduction in systolic aortic blood pressure was observed. The diastolic blood pressure indicates how much pressure the heart exerts on the walls of the arteries in between beats and can therefore be used as marker of vascular function. For this reason, diastolic blood pressure may be more important than systolic blood pressure when studying the relaxing effects of music, and appears consistent in view of our results. Left ventricular ejection time, which indicates the length of the systole, was extended by both interventions, physiologically according to the reduction in heart rate. In addition to objectively measurable cardiovascular parameters, psychological outcomes were also recorded in this study using VAS. Both interventions were able to improve psychological outcomes, which was particularly evident in an increase in emotional well-being. This has also been described in a systematic review [34]. In addition, both interventions led to a reduction in fatigue, with a more pronounced effect for 432 Hz. These results are also consistent with previous studies in which music interventions (both prerecorded music and participating in live music) could relieve cancer-related fatigue [35]. Indeed, only short-term effects are documented within this trial and studies with more frequent interventions are warranted to assess long-term impact. Furthermore, a relief of anxiety and stress was also achieved in a large number of study participants, although the majority showed no increased VAS scores even before the intervention. This suggests either that the patients are receiving appropriate care and that psychological problems are also adequately addressed during therapy, or that only patients with correspondingly low scores participated in the study. Interestingly, other 432 Hz studies carried out to date also found no relevant difference in terms of subjective stress reduction [23, 24, 36], while there was a significant improvement in sleep scores [36] and lower salivary cortisol levels [37]. The effects of 432 Hz music do not appear to be perceivable for the majority of patients, but several objective results obtained from different studies indicate an effect on the cardiovascular system. This is consistent with the personal assessment of our study participants, about two thirds of whom stated that they had noticed a difference and slightly more than half preferred the 432 Hz intervention. Our study thus builds on the existing data and corroborates and expands the previous findings. It should be mentioned that our study population of 43 participants is the largest compared to the existing studies. In addition, other cardiovascular parameters were recorded in comparison to the standard vital parameters. An overview of previous studies compared to the current study investigating the effects of 432 Hz music compared to 440/443 Hz can be found in the **Supplemental Table** in the appendix.

### Strengths and limitations

The present study compared sound interventions tuned to 432 Hz and 443 Hz, the latter representing the concert pitch in German professional orchestras. Whether the beneficial effects on cardiovascular parameters and psychological outcomes are actually attributable to the specific tuning of 432 Hz or whether lower tunings are generally perceived as more pleasant cannot be answered with certainty on the basis of the available data. Further studies should be performed to address this question, comparing for instance 432 Hz music with an even lower tuning (e.g., 415 Hz, which is often used for baroque music). Individuals with absolute perception („absolute listeners“) may experience difficulties with 432 Hz music, as they cannot reliably assign the tones they hear to a pitch. It is therefore unclear whether a sound intervention tuned to 432 Hz also exerts beneficial effects on absolute listeners. Since no relevant difference was found in the improvement of subjective parameters, a strength of the study is to combine objective measures such as cardiovascular parameters with psychological outcomes to obtain a comprehensive assessment. In addition, music played live via a body monochord was used. A monochord compared to recorded music offers the advantage of a more vivid mixture of overtones, which could play a special role in mode of action of 432 Hz music (“oscillation ratio”).

## Conclusion

This study compared the differential effects of sound interventions tuned to 432–443 Hz on cardiovascular parameters and psychological outcomes in cancer patients. While both interventions improved psychological outcomes, a greater influence on cardiovascular parameters was observed with 432 Hz, but not significantly different compared to 443 Hz. To what extent the positive effects are actually attributable to the specific tuning of 432 Hz or whether lower tunings are generally perceived as more pleasant cannot be answered with certainty on the basis of the available data. The present study confirms and corroborates previous results that 432 Hz music exerts beneficial effects, in particular on cardiovascular parameters, whose increase is associated with an increased stress level.

## Appendix

### Measurement protocol of cardiovascular parameters

- Initial Rest Phase: Patients were first asked to rest in a lying position for 10 min to stabilize their vital signs.
- Pre-Intervention Measurement: After the initial 10-minute rest phase, the cardiovascular parameters were measured using the VascAssist 2 device. This measurement took approximately 10 min.
- Intervention Phase: Following the pre-intervention measurement, the patients underwent a 15-minute music intervention with either 432–443 Hz music.
- Post-Intervention Rest Phase: After the intervention, patients rested again in a lying position for another 10 min.
- Post-Intervention Measurement: The cardiovascular parameters were then measured again using the VascAssist 2 device.

## Electronic supplementary material

Below is the link to the electronic supplementary material.

Supplementary Material 1

Supplementary Material 2

## Data Availability

The data that support the findings of this study will be made available from the corresponding author upon reasonable request.

## References

[1] Rosenberg RE. Perfect pitch: 432 hz music and the Promise of frequency. J Popular Music Stud 1 März. 2021;33(1):137–54.

[2] Kaye GWC. International Standard of Concert Pitch. Nat 1 Mai. 1939;143(3630):905–6.

[3] Stamper GC, Johnson TA. Auditory function in Normal-Hearing, noise-exposed human ears. Ear Hear April. 2015;36(2):172. 10.1097/AUD.0000000000000107 PMC4374361 25350405

[4] Picton T. Hearing in Time: evoked potential studies of temporal Processing. Ear Hear August. 2013;34(4):385. 10.1097/AUD.0b013e31827ada02 24005840

[5] Calamassi D, Pomponi GP. Music tuned to 440 hz Versus 432 hz and the Health effects: a double-blind cross-over pilot study. Explore (NY). 2019;15(4):283–90. 31031095 10.1016/j.explore.2019.04.001

[6] Dubey P, Kumar Y, Singh R, Jha K, Kumar R. Effect of music of specific frequency upon the sleep architecture and electroencephalographic pattern of individuals with delayed sleep latency: a daytime nap study. J Family Med Prim Care Dezember. 2019;8(12):3915–9. 10.4103/jfmpc.jfmpc_575_19 PMC6924256 31879635

[7] Gold C, Erkkilä J, Bonde LO, Trondalen G, Maratos A, Crawford MJ. Music Therapy or Music Medicine? Psychother Psychosom 30 Juni. 2011;80(5):304. 10.1159/000323166 21720190

[8] J JCR. The Distinction that exists between Music Therapy and Music Medicine. Blog [Internet]. 1. Januar 2024 [zitiert 19. Dezember 2024]; Verfügbar unter: https://www.academia.edu/125753587/The_Distinction_that_exists_between_Music_Therapy_and_Music_Medicine

[9] Calamassi D, Lucicesare A, Pomponi GP, Bambi S. Music tuned to 432 hz versus music tuned to 440 hz for improving sleep in patients with spinal cord injuries: a double-blind cross-over pilot study. Acta Biomed 30 November. 2020;91(12–S):e2020008. 10.23750/abm.v91i12-S.10755 PMC8023109 33263352

[10] Erdal B, Tepe YK, Çelik S, Güçyetmez B, Çiğdem B, Topaktaş S. The magic of frequencies – 432 hz vs. 440 hz: do cheerful and sad music tuned to different frequencies cause different effects on human psychophysiology? A neuropsychology study on music and emotions: Frekansların sihri – 432 hz 440 hz’. J Hum Sci 17 Januar. 2021;18(1):12–33. e karşı: Ayrı frekanslara göre akortlanmış neşeli ve hüzünlü müzikler insan psikofizyolojisi üzerinde farklı etkiler yaratır mı? Müzik ve duygular üzerine bir nöropsikoloji araştırması.

[11] Spielberger CD, Vagg PR. Psychometric properties of the STAI: a reply to Ramanaiah, Franzen, and Schill. J Personality Assess 1 Februar. 1984;48(1):95–7. 10.1207/s15327752jpa4801_16 6707862

[12] Scott NW, Fayers P, Aaronson NK, Bottomley A, de Graeff A. Groenvold M, EORTC QLQ-C30 reference values manual. Juli 2008 [zitiert 22. Oktober 2021]; Verfügbar unter: https://abdn.pure.elsevier.com/en/publications/eortc-qlq-c30-reference-values-manual

[13] Linhuber C. Himmel und Erde - Klangkörper [Internet]. [zitiert 21. Oktober 2021]. Verfügbar unter: http://www.klangkoerper.de/himmel-und-erde.html

[14] Gäbel C, Garrido N, Koenig J, Hillecke TK, Warth M. Effects of Monochord Music on Heart Rate Variability and Self-Reports of Relaxation in healthy adults. Complement Med Res 3 Februar. 2017;24(2):97–103. 10.1159/000455133 28192781

[15] Pérez-Eizaguirre M, Vergara-Moragues E. Music therapy interventions in Palliative Care: a systematic review. J Palliat Care 1 Juli. 2021;36(3):194–205. 10.1177/0825859720957803 32928042

[16] Sigl M, Winter L, Schumacher G, Helmke SC, Shchetynska-Marinova T, Amendt K et al. Comparison of Functional and Morphological Estimates of Vascular Age. in vivo. 2023. 10.21873/invivo.13317 PMC10500512 37652489

[17] Li Z, Snieder H, Su S, Ding X, Thayer JF, Treiber FA. u. a. A longitudinal study in youth of heart rate variability at rest and in response to stress. Int J Psychophysiol 1 September. 2009;73(3):212–7. 10.1016/j.ijpsycho.2009.03.002 PMC2719684 19285108

[18] Laux L, Glanzmann P, Schaffner P, Spielberger CD. State-trate anxiety inventory–German version. Weinheim: Beltz Test GmbH; 1981.

[19] Levitin DJ, Rogers SE. Absolute pitch: perception, coding, and controversies. Trends Cogn Sci 1 Januar. 2005;9(1):26–33. 10.1016/j.tics.2004.11.007 15639438

[20] Carden J, Cline T. Absolute pitch: myths, evidence and relevance to music education and performance. Psychol Music 1 November. 2019;47(6):890–901.

[21] Wong YK, Lui KFH, Yip KHM, Wong ACN. Is it impossible to acquire absolute pitch in adulthood? [Internet]. bioRxiv; 2019 [zitiert 2. Februar 2024]. S. 355933. Verfügbar unter: https://www.biorxiv.org/content/10.1101/355933v2

[22] Kopka M. The influence of music tuned to 440 Hz & 432 Hz on the perceived arousal. In: Grünewald-Schukalla L, Jóri A, Schwetter H, Herausgeber. Musik & Marken [Internet]. Wiesbaden: Springer Fachmedien; 2022 [zitiert 29. Januar 2024]. S. 227–45. (Jahrbuch für Musikwirtschafts- und Musikkulturforschung). Verfügbar unter: 10.1007/978-3-658-36472-4_10

[23] Calamassi D, Pomponi GP. Music tuned to 440 hz Versus 432 hz and the Health effects: a double-blind cross-over pilot study. EXPLORE 1 Juli. 2019;15(4):283–90. 10.1016/j.explore.2019.04.001 31031095

[24] Calamassi D, Li Vigni ML, Fumagalli C, Gheri F, Pomponi GP, Bambi S. Listening to music tuned to 440 hz versus 432 hz to reduce anxiety and stress in emergency nurses during the Covid-19 pandemic: a double-blind, randomized controlled pilot study. Acta Biomed. 2022;93(Suppl 2):e2022149. 35545982 10.23750/abm.v93iS2.12915 PMC9534204

[25] Zhou X, Ma Z, Zhang L, Zhou S, Wang J, Wang B. u. a. heart rate variability in the prediction of survival in patients with cancer: a systematic review and meta-analysis. J Psychosom Res 1 Oktober. 2016;89:20–5. 10.1016/j.jpsychores.2016.08.004 27663106

[26] Kloter E, Barrueto K, Klein SD, Scholkmann F, Wolf U. Heart Rate Variability as a prognostic factor for Cancer survival – a systematic review. Front Physiol [Internet]. 2018. 10.3389/fphys.2018.00623. [zitiert 16. Januar 2024];9. Verfügbar unter:. https://www.frontiersin.org/articles/. 29896113 10.3389/fphys.2018.00623 PMC5986915

[27] Nabeel PM, Kiran VR, Joseph J, Abhidev VV, Sivaprakasam M. Local pulse Wave Velocity: theory, methods, advancements, and clinical applications. IEEE Rev Biomed Eng. 2020;13:74–112. 31369386 10.1109/RBME.2019.2931587

[28] Vlachopoulos C, Aznaouridis K, O’Rourke MF, Safar ME, Baou K, Stefanadis C. Prediction of cardiovascular events and all-cause mortality with central haemodynamics: a systematic review and meta-analysis. Eur Heart J August. 2010;31(15):1865–71. 10.1093/eurheartj/ehq024 20197424

[29] Hohneck A, Reyser C, Merx K, Weingärtner S, Mavratzas A, Schumacher G. u. a. Differential effects of Sound intervention and rest on Cardiovascular parameters in Cancer patients: a randomized cross-over trial. Integr Cancer Ther Januar. 2021;20:153473542199523. 10.1177/1534735421995239 PMC8024455 33813930

[30] Hohneck A, Reyser C, Usselmann R, Heinemann L, Weingaertner S, Reckling H. Hemodynamic and stress response after sound intervention with different Headphone systems: a double-blind randomized study in healthy volunteers working in the Health Care Sector. Journal of Integrative and complementary medicine [Internet]. 11. Oktober 2023 [zitiert 11. Dezember 2023]; Verfügbar unter: https://www.liebertpub.com/doi/10.1089/jicm.2022.0757 10.1089/jicm.2022.0757 37819750

[31] Aggelakas A, Vlachopoulos C, Xaplanteris P, Synodinos A, Kardara D, Abdelrasoul M. u. a. music to my ears, heart and aorta: the effect of music listening on arterial stiffness and aortic hemodynamics of young, healthy volunteers. Eur Heart J 1 August. 2013;34(suppl1):P5150.

[32] Vlachopoulos C, Aggelakas A, Ioakeimidis N, Xaplanteris P, Terentes-Printzios D, Abdelrasoul M. u. a. music decreases aortic stiffness and wave reflections. Atherosclerosis Mai. 2015;240(1):184–9. 10.1016/j.atherosclerosis.2015.03.010 25796036

[33] Groenewegen K, den Ruijter H, Pasterkamp G, Polak J, Bots M, Peters SA. Vascular age to determine cardiovascular disease risk: a systematic review of its concepts, definitions, and clinical applications. Eur J Prev Cardiol 1 Februar. 2016;23(3):264–74. 10.1177/2047487314566999 25609227

[34] Li Y, Xing X, Shi X, Yan P, Chen Y, Li M. u. a. The effectiveness of music therapy for patients with cancer: a systematic review and meta-analysis. J Adv Nurs. 2020;76(5):1111–23. 32017183 10.1111/jan.14313

[35] Qi Y, Lin L, Dong B, Xu E, Bao Z, Qi J. u. a. music interventions can alleviate cancer-related fatigue: a metaanalysis. Support Care Cancer 1 Juli. 2021;29(7):3461–70. 10.1007/s00520-021-05986-4 33481115

[36] Calamassi D, Lucicesare A, Pomponi GP, Bambi S. Music tuned to 432 hz versus music tuned to 440 hz for improving sleep in patients with spinal cord injuries: a double-blind cross-over pilot study. Acta Biomed. 2020;91(Suppl 12):e2020008. 33263352 10.23750/abm.v91i12-S.10755 PMC8023109

[37] Aravena PC, Almonacid C, Mancilla MI. Effect of music at 432 hz and 440 hz on dental anxiety and salivary cortisol levels in patients undergoing tooth extraction: a randomized clinical trial. J Appl Oral Sci 11 Mai. 2020;28:e20190601. 10.1590/1678-7757-2019-0601 PMC7213780 32401941

