# Supplementary material for: Differential effects of sound interventions tuned to 432 Hz or 443 Hz on cardiovascular parameters in cancer patients: a randomized cross-over trial
Source: BMC Complement Med Ther. 2025 Jan 22;25:18. doi: 10.1186/s12906-025-04758-5 (PMC11755923; doi:10.1186/s12906-025-04758-5)
Supplement: Supplementary file 1 — Supplementary Material 1 [file 12906_2025_4758_MOESM1_ESM.docx]

**Supplemental Table. Cross trial comparison of studies investigating the effects of 432 Hz music compared to 440/443 Hz**

|  | Study population | Design | Outcomes | Results | |
| --- | --- | --- | --- | --- | --- |
|  |  |  |  | 432 Hz | 440/443 Hz |
| Calamassi D, Pomponi GP 2019 | **33** healthy volunteers | Cross-over,  432 Hz vs 440 Hz | Vitals (HR, BP, RR) | significant HR reduction, slight decrease of BP and RR | slight HR reduction, slight increase in BP, no change in RR |
|  |  |  | Perceptions | significant increase in attention level and general satisfaction | no difference |
| Calamassi D et al. 2020 | **12** patients with spinal injuries | Cross-over,  432 Hz vs 440 Hz | Sleep Scale for Medical Study | significant improvement in sleep scores | no difference |
|  |  |  | Perceived Stress Scale | slight decrease in stress | slight decrease in stress |
| Aravena PC et al. 2020 | 42 patients undergoing tooth extraction | 3-arm-comparison:  432 Hz (n = **15**) vs 440 Hz (n = 15) vs control (n = 12) | CORAH Dental Anxiety Scale | significant reduction in anxiety | significant reduction in anxiety |
|  |  |  | Salivary cortisol levels | no intragroup difference, but significantly lower compared to control and 440 Hz | no difference |
| Calamassi et al. 2022 | 54 healthcare providers | 3-arm-comparison:  432 Hz (n = **18**) vs 440 Hz (n = 18) vs control (n = 18) | State-Trait Anxiety Inventory (STAI) | significant reduction in STAI scores | significant reduction in STAI scores |
|  |  |  | Vitals (HR, BP, RR) | Trend towards lower HR, significant reduction of RR and BP | no difference |
|  |  |  | Likert scales (pain, productivity) | no difference | no difference |
| **Current study** | | | | | |
| Hohneck et al. | **43** patients with cancer | Cross-over,  432 Hz vs 443 Hz | Cardiovascular parameters   - HR, BP - HRV - resistance/stiffness, PWV | - significant reduction in HR, systolic and diastolic BP - significant increase in HRV - significant reduction in resistance/stiffness and PWV | - significant reduction in HR and systolic BP, no change in diastolic BP - no change in HRV - no change in resistance/stiffness or PWV |
|  |  |  | VAS (emotional well-being, anxiety, stress, fatigue) | significant improvement in all parameters | significant improvement in all parameters |
