# Supplementary material for: Differential effects of sound interventions tuned to 432 Hz or 443 Hz on cardiovascular parameters in cancer patients: a randomized cross-over trial
Source: BMC Complement Med Ther. 2025 Jan 22;25:18. doi: 10.1186/s12906-025-04758-5 (PMC11755923; doi:10.1186/s12906-025-04758-5)
Supplement: Supplementary file 2 — Supplementary Material 2 [file 12906_2025_4758_MOESM2_ESM.docx]

**SUPPLEMENTALS**

**Supplemental Table 1. Cancer types, stages and treatment**

|  | | **All patients (N = 43)** |
| --- | --- | --- |
| **Cancer types** | |  |
|  | Breast cancer | 21 (48.8) |
|  | Colorectal cancer | 15 (34.9) |
|  | HPB | 3 (7.0) |
|  | Lymphoma | 2 (4.7) |
|  | Others | 2 (4.7) |
| **Advanced disease stage** | | 12 (27.9) |
| **Anticancer treatment** | |  |
|  | Chemotherapy | 35 (81.4) |
|  | Immunotherapy | 28 (65.1) |
|  | Surgery | 27 (62.8) |
|  | Radiotherapy | 20 (46.5) |
|  | Hormone therapy | 5 (11.6) |
